# Treatment Patterns and Clinical Outcomes Among Patients Receiving Palbociclib Combinations for Hormone Receptor–Positive (HR+)/Human Epidermal Growth Factor Receptor 2–Negative (HER2−) Advanced/Metastatic Breast Cancer in the Arabian Gulf Region: A Retrospective Study (TREASURE Study)

**DOI:** 10.1155/ijbc/3477817

**Published:** 2026-09-22

**Authors:** Emad Dawoud, Francois Calaud, Ashraf Awad Fadlelseid, Nafisa Abdelhafeez, Sharif Kullab, Muhammad Farooq Latif, Mohamed Omara, Syed Tirmazy, Ahmed Hashiesh, Ali AlJabban, Shaheenah Dawood, Meteb Al-Foheidi

**Affiliations:** ^1^ Tawam Hospital, Al Ain, UAE, tawamhospital.ae; ^2^ Hamad Medical Corporation, Doha, Qatar, hamad.qa; ^3^ National Guard Hospital, Riyadh, Saudi Arabia, mngha.med.sa; ^4^ King Saud University, Riyadh, Saudi Arabia, ksu.edu.sa; ^5^ Dubai Hospital, Dubai, UAE, dohms.gov; ^6^ Pfizer Inc., Riyadh, Saudi Arabia, pfizer.com; ^7^ Pfizer Inc., Dubai, Saudi Arabia, pfizer.com; ^8^ Mediclinic Hospital, Dubai, UAE; ^9^ National Guard Hospital, Jeddah, Saudi Arabia, mngha.med.sa

**Keywords:** Arabian Gulf countries, HR+/HER2−, metastatic breast cancer, palbociclib, treatment patterns

## Abstract

**Background:**

Palbociclib, a cyclin‐dependent kinase 4/6 (CDK4/6) inhibitor, has been approved for advanced/metastatic breast cancer (ABC) treatment. However, real‐world data on its use in the Arabian Gulf region are limited.

**Methods:**

The TREASURE study, a retrospective, multicenter study, evaluated patients with hormone receptor–positive/human epidermal growth factor receptor 2–negative (HR+/HER2−) ABC treated with palbociclib + endocrine therapy (ET) in the Arabian Gulf. Data were abstracted from the medical records of eligible patients from 7 selected sites across Arabian Gulf countries. The study evaluated patient demographics, clinical characteristics, treatment patterns, and clinical outcomes in those who initiated palbociclib + ET from April 2015 to September 2019.

**Results:**

The study comprised 258 patients (median age: 53.7 years) who received palbociclib in the first‐line (1L) (*n* = 93, 36.0%), second‐line (2L) (*n* = 61, 23.6%), or third‐line and beyond (3L+) (*n* = 30, 11.6%). Treatment regimens included palbociclib plus aromatase inhibitors (*n* = 142, 55.0%) and palbociclib with fulvestrant (*n* = 120, 46.5%). The starting dose of palbociclib was 125 mg for 228 patients (88.4%). The median duration of follow‐up was 16.8 months. Global median real‐world progression‐free survival (rwPFS) was 26.5 months (1L: 40.3, 2L: 18.5, 3L+: 17.2). The rwPFS rates at 12, 24, and 36 months were as follows: 1L—84.5%, 72.8%, and 58.4%; 2L—69.8%, 38.8%, and 16.2%; 3L+—80.0%, 42.3%, and 23.5%.

**Conclusion:**

This study provides initial data on palbociclib′s real‐world treatment management and effectiveness in the Arabian Gulf region, with results consistent with those reported in clinical trials and other real‐world evidence studies. Larger studies with more patients are needed to further assess long‐term clinical outcomes in this population.

**Trial Registration:** ClinicalTrials.gov identifier: NCT04916509

## 1. Introduction

In 2020, approximately 2.3 million women worldwide were newly diagnosed with breast cancer (BC). BC was responsible for approximately 685,000 female deaths in the same year, ranking it as the fifth leading cause of cancer‐related mortality [1]. At the time of diagnosis, about 30% of patients with BC have regional lymph node involvement, whereas another 6% present with distant metastases [2]. An additional 20%–30% of patients with early BC will develop metastatic disease. Among BC subtypes, the hormone receptor–positive and HER2‐negative (HR+/HER2−) subtype is the most prevalent, accounting for approximately 70% of all BC cases [3]. Approximately 60%–75% of patients with advanced/metastatic breast cancer (ABC), which includes both locally advanced BC and metastatic BC, are diagnosed with the HR+/HER2− subtype [4, 5].

In Arab countries, patients often present with ABC at the time of diagnosis [6]. Data from registries across five countries in the Middle East and North Africa (MENA) region (Algeria, Bahrain, Morocco, Oman, and the United Arab Emirates) report that early‐stage disease (Stages I and II) represented 65.4% of cases, with the lowest percentage in Oman (57.9%) and the highest in Algeria (83.3%). Distant metastatic disease (Stage IV) at initial diagnosis was present in 9.9% of patients. Of note, in both Bahrain and Algeria, fewer than 0.5% of women were initially diagnosed with distant metastatic disease [7]. Axillary lymph node involvement is frequent, with prevalence rates up to 79% in Yemen and Saudi Arabia and 50% in Bahrain [8–10]. In the United Arab Emirates, 46.2% of BC cases are diagnosed at Stage IV [6]. BC in Arab women typically exhibits HR‐negative status, poor differentiation in histopathology, and higher HER2 expression, although there is notable variability in molecular subtypes across different Arab populations [6]. In Saudi Arabia, for instance, 43% of BC cases are unclassified/penta‐negative (triple‐negative tumors not exhibiting basal markers [cytokeratin 5/6‐ and epidermal growth factor receptor‐negative]) [11]. Also, a recent study in the United Arab Emirates highlighted the significant cost burden of BC: 84% of the disease burden was attributable to BC‐specific costs [12].

Endocrine therapy (ET) remains the mainstay of treatment for patients who are HR+ [13]. These therapies have long been at the forefront of HR+ ABC management. The development of resistance to ET has led to endocrine drug combinations, resulting in a transformation in the landscape of ABC management [14, 15]. Palbociclib was the first approved cyclin‐dependent kinase 4/6 (CDK4/6) inhibitor and has played a pivotal role in this evolving landscape. It was approved by the US Food and Drug Administration for use in combination with aromatase inhibitors (AIs) as an initial endocrine‐based therapy or in combination with fulvestrant for postmenopausal women who have previously undergone ET [16, 17].

Palbociclib in combination with ET improved progression‐free survival (PFS) and demonstrated an acceptable safety profile, as observed in clinical trials. This benefit was initially established in the PALOMA‐1 Phase 2 trial [18] and later confirmed in the randomized PALOMA‐2 Phase 3 study, which showed improvement in PFS in patients receiving palbociclib in combination with letrozole as 1L treatment compared with placebo plus letrozole (median PFS: 24.8 months for palbociclib–letrozole vs. 14.5 months for placebo–letrozole; hazard ratio [HR] = 0.58; *p* < 0.001) [19]. However, the final overall survival (OS) analysis, after a median follow‐up of approximately 90 months, showed no statistically significant difference between treatment arms (median OS: 53.9 months for palbociclib–letrozole vs. 51.2 months for placebo–letrozole; HR = 0.96, 95% confidence interval [CI]: 0.78–1.18; *p* = 0.34) [20].

The Phase 3 PALOMA‐3 trial showed that palbociclib in combination with fulvestrant prolonged PFS in patients whose disease progressed or relapsed after ET. The median PFS was 9.5 months for palbociclib + fulvestrant versus 4.6 months for placebo + fulvestrant (HR = 0.46; 95% CI, 0.36–0.59; *p* < 0.001). Moreover, palbociclib + fulvestrant showed a clinically meaningful improvement in OS, with a median OS of 34.9 months versus 28.0 months for placebo + fulvestrant (HR = 0.81; 95% CI, 0.64–1.01; *p* = 0.09). Although the OS difference did not reach statistical significance, the benefit was sustained for over 6 years of follow‐up, with the most pronounced improvement in patients with endocrine‐sensitive disease and those without prior chemotherapy [21].

The Phase II PARSIFAL trial compared fulvestrant and letrozole, both combined with palbociclib, in endocrine‐sensitive HR+/HER2− ABC. The study found no significant difference in PFS between the groups, confirming letrozole as the preferred endocrine partner, although fulvestrant remains an option for patients intolerant to AIs and/or patients pretreated with AI [22]. The extended follow‐up (PARSIFAL‐LONG) reported a combined median OS of 65.4 months, consistent with expected outcomes for CDK4/6 inhibitors in 1L treatment [23–25]. These findings align with PALOMA‐2, further reinforcing the role of palbociclib‐based regimens in HR+/HER2− ABC.

Although randomized controlled trials (RCTs) serve as the gold standard for establishing the efficacy and safety of new drugs, it is important to acknowledge that the patient populations enrolled in these trials often do not represent the diverse range of patients encountered in real‐world clinical practice. Strict inclusion criteria and trial protocols can limit the representation of various patient demographics. Real‐world studies have consistently revealed clinically relevant distinctions between RCT populations and real‐world patients with BC, including differences in age, tumor size, and visceral metastasis, among other differences [26, 27].

Therefore, real‐world studies play a crucial role in assessing the impact of new therapies in real‐world patient populations. Furthermore, these studies offer significant insights for healthcare professionals regarding the management of real‐world, unselected patient populations. In the field of oncology, as new therapies are introduced, there is growing interest regarding the reproducibility of RCT findings within real‐world populations. Real‐world evidence (RWE) has assumed a more significant role in health care decision‐making [28]. A number of country‐specific analyses have been conducted globally to examine the characteristics of ABC and medication performance across different patient populations, such as Ibrance Real World Insights (IRIS) [29–37].

RWE on palbociclib has been generated mainly in Western populations, with limited data from the MENA region. A recent systematic scoping review found that current knowledge of the real‐world effectiveness, safety, and treatment patterns of palbociclib derives mainly from studies conducted in North America, Europe, Australia, and New Zealand, and that recent regulatory requirements on equity, diversity, and inclusion (EDI) may improve representation of patients from underrepresented regions in future research [38]. Few peer‐reviewed real‐world reports of palbociclib are available from MENA countries, and these are limited to single‐country cohorts [39, 40]. Data from the Arabian Gulf are particularly lacking. The aim of this study was to describe the demographics, clinical characteristics, treatment patterns, and clinical outcomes of patients receiving palbociclib for HR+/HER2− ABC, in line with the approved indication, across the Arabian Gulf region.

## 2. Materials and Methods

### 2.1. Study Design

This was a retrospective, multicenter, multicountry, noninterventional study that is aimed at describing the demographics, clinical characteristics, treatment patterns, and clinical outcomes of patients receiving palbociclib for the treatment of HR+/HER2− ABC. This study leveraged a retrospective review of medical records in select Arabian Gulf region countries (Saudi Arabia, the United Arab Emirates, and Qatar).

Eligible physicians were identified based on an initial survey. To be eligible, physicians had to have treated at least 4 patients with HR+/HER2− ABC with palbociclib, in line with the locally approved indication, and had to be willing to participate in the study and complete the electronic case report forms (eCRFs) within the data collection period. Eligible patients were aged ≥ 18 years and had a confirmed diagnosis of HR+/HER2− ABC. To ensure sufficient follow‐up for outcome assessments, a minimum of 6 months of potential follow‐up since the initiation of palbociclib was required (patients who discontinued treatment due to disease progression or any other reasons, including death within 6 months, were included for analysis). Patients with prior or current enrollment in an interventional clinical trial were excluded.

### 2.2. Data Sources and Abstraction

Each physician completed eCRFs by retrospectively abstracting medical data for patients who met the inclusion and exclusion criteria. Data were collected from seven selected sites across the Arabian Gulf region (Saudi Arabia: three sites, United Arab Emirates: three sites, and Qatar: one site) for patients who initiated treatment with palbociclib between April 2015 and September 2019. Data were abstracted over an observational period from April 2015 to December 2022. The selected sites were from diverse health care settings (e.g., university and community hospitals). Each physician completed up to 50 eCRFs from respective consecutive patients. Patient demographics, clinical characteristics, comorbid conditions, concomitant medications, complete BC treatment history, and clinical outcomes (real‐world progression‐free survival [rwPFS] and OS) were collected.

### 2.3. Endocrine Sensitivity Classification

Endocrine sensitivity was defined as patients who had never received ET for early breast cancer (EBC), those who relapsed ≥ 12 months after completion of adjuvant ET, or those diagnosed with de novo stage IV BC. Primary endocrine‐resistant disease was defined as relapse while on the first 2 years of adjuvant ET, or disease progression within the first 6 months of 1L ET for advanced BC while on ET. Secondary (acquired) resistance was defined as relapse while on adjuvant ET but after the first 2 years, or relapse within 12 months of completing adjuvant ET, or disease progression ≥ 6 months after initiating ET for advanced BC while on ET. For patients receiving palbociclib in 2L, 3L, or later, resistance classification was based on their most recent prior ET before starting palbociclib in the metastatic setting.

### 2.4. Outcome Assessment

Tumor response and disease progression were determined by the treating physicians at each site as part of routine clinical practice, based on the clinical and radiological information available in the medical record at the time of assessment. The choice and frequency of imaging assessments, and the imaging modality used, followed each site′s local standard of care.

### 2.5. Statistical Analysis

This was a descriptive study, and as such, no formal hypothesis testing was conducted. Summary statistics for continuous variables included the number of observations, means, standard deviations, medians, and ranges. Summary statistics for categorical data included the number of observations, counts, and percentages. Time‐to‐event outcomes were estimated using Kaplan–Meier estimates at 12, 18, 24, and 36 months.

Kaplan–Meier estimates of median rwPFS were calculated, with pointwise 95% confidence intervals estimated using Greenwood′s formula with the log–log transformation. rwPFS was defined as the time from the initiation of palbociclib treatment until the first reported disease progression or death due to any cause, whichever occurred first. Patients were censored at the last follow‐up or at the end of the observation period. OS was defined as the time from palbociclib initiation to death, censoring at last follow‐up or at the end of the observation period. Further, rwPFS and OS were assessed in patients with known outcomes and lines of treatment. The analyses were performed for the overall population, as well as by lines of treatment.

## 3. Results

### 3.1. Study Population

Between April 2015 and September 2019, data of 258 patients (full cohort) were abstracted. At BC diagnosis, 143 patients (55.4%) had ABC, whereas 101 (39.1%) had EBC and then experienced recurrence. At metastatic diagnosis, bone was the most common metastatic site, affecting 172 patients (66.7%), followed by lymph nodes (*n* = 92; 35.7%), lung (*n* = 78; 30.2%), and liver (*n* = 66; 25.6%; metastatic locations are nonexclusive).

**Figure 1:**
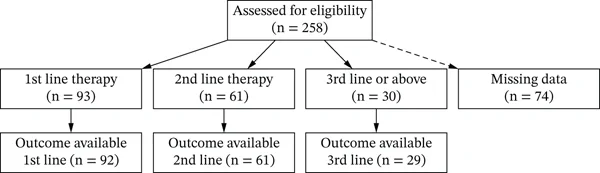
Patient flow diagram.

Palbociclib was administered as 1L treatment for ABC in 93 patients (36.0%), 2L in 61 (23.6%), and 3L in 30 (11.6%). There were 74 cases (28.7%) where the line of treatment could not be determined due to missing data (Figure 1).

Figure 1 describes the patient allocation.

The median age at initiation of palbociclib was 53.7 years (interquartile range [IQR]: 44.1–63.3). Premenopausal women comprised 39.2% (*n* = 101) of the patients, whereas 53.5% (*n* = 138) were postmenopausal. Menopausal status was missing for 7.4% (*n* = 19) of the patients.

At palbociclib initiation, 46 patients (17.8%) were considered to have endocrine‐sensitive disease, as assessed by the treating physician. A total of 16 patients (6.2%) were classified as having primary endocrine‐resistant disease, and 10 patients (3.9%) were classified as having secondary (acquired) resistance. A total of 51 patients (19.8%) had unknown endocrine sensitivity status, and information for endocrine status was missing for 135 patients (52.3%).

### 3.2. Demographic and Clinical Characteristics of Patients by Line of Treatment

The median age by line of treatment was 53.1 years (IQR: 43.5–62.8) for 1L, 55.8 years (IQR: 45.3–62.2) for 2L, and 59.0 years (IQR: 45.5–68.7) for 3L+.

Endocrine sensitivity was noted in 30.1% (*n* = 28) of 1L, 18.0% (*n* = 11) of 2L, and 20% (*n* = 6) of 3L+ patients. Primary resistance rates were 1.1% (*n* = 1) for 1L, 23.0% (*n* = 14) for 2L, and 0% for 3L+. Secondary resistance rates were 2.2% (*n* = 2) for 1L, 8.2% (*n* = 5) for 2L, and 10.0% (*n* = 3) for 3L+. Endocrine resistance data were unknown or missing in 72.1% overall.

The median follow‐up duration by line of treatment was 20.4 months (IQR: 9.6–27.6) for 1L, 12.0 months (IQR: 7.2–25.2) for 2L, and 16.8 months (IQR: 9.6–25.2) for 3L+.

Patient characteristics are summarized in Table 1.

**Table 1:** Demographic and clinical characteristics of patients—overall and by line of treatment.

Variable	Overall	Line of treatment			
		1L	2L	3L+	Missing
*N* patients	258 (100%)	93 (36.0%)	61 (23.6%)	30 (11.6%)	74 (28.7%)
(% of cohort)					
Age at initiation of palbociclib (years)					
Mean (SD)	54.1 (12.26)	53.6 (12.02)	54.3 (12.05)	57.1 (13.07)	53.5 (12.47)
Median	53.7	53.1	55.8	59.0	51.4
IQR	(44.1, 63.3)	(43.5, 62.8)	(45.3, 62.2)	(45.5, 68.7)	(44.0, 62.7)
Min, Max	23.2, 82.8	26.9, 80.9	33.5, 82.8	33.1, 77.4	23.2, 80.3
Missing/NA	1	1	0	0	0
Family history of BC (*n* [%])					
No	176 (68.2%)	59 (63.4%)	39 (63.9%)	19 (63.3%)	59 (79.7%)
Yes	51 (19.8%)	24 (25.8%)	12 (19.7%)	5 (16.7%)	10 (13.5%)
Missing	31 (12.0%)	10 (10.8%)	10 (16.4%)	6 (20.0%)	5 (6.8%)
Stage of BC at diagnosis (*n* [%])					
ABC	143 (55.4%)	67 (72.0%)	31 (50.8%)	21 (70.0%)	25 (33.8%)
EBC	101 (39.1%)	25 (26.9%)	30 (49.2%)	8 (26.7%)	37 (50.0%)
Missing	14 (5.4%)	1 (1.1%)	0	1 (3.3%)	12 (16.2%)
Menopausal status at initiation of palbociclib (*n* [%])					
Premenopausal	101 (39.2%)	38 (40.9%)	24 (39.3%)	9 (30.0%)	30 (40.5%)
Postmenopausal	138 (53.5%)	47 (50.5%)	29 (47.5%)	19 (63.3%)	43 (58.1%)
Missing/NA	19 (7.4%)	8 (8.6%)	8 (13.1%)	2 (6.7%)	1 (1.4%)
Age at menopause (years)					
Mean (SD)	49.7 (4.47)	47.5 (6.16)	51.4 (2.07)	48.4 (2.07)	50.0 (.)
Median	50	50	50.5	49	50
IQR	(49.0, 53.0)	(46.0, 50.5)	(50.0, 53.0)	(48.0, 50.0)	(50.0, 50.0)
Min, Max	32.0, 55.0	32.0, 55.0	49.0, 55.0	45.0, 50.0	50.0, 50.0
Missing/NA	221	81	53	25	0
ECOG performance status at initiation of palbociclib (*n* [%])					
0	30 (11.6%)	16 (17.2%)	3 (4.9%)	2 (6.7%)	9 (12.2%)
1	58 (22.5%)	22 (23.7%)	26 (42.6%)	5 (16.7%)	5 (6.7%)
2+	8 (3.1%)	4 (4.3%)	1 (1.6%)	1 (3.3%)	2 (2.7%)
Missing	162 (62.8%)	51 (54.8%)	31 (50.8%)	22 (73.3%)	58 (78.4%)
Sites of metastasis^a^ (*n* [%])					
Bone	172 (66.7%)	63 (67.7%)	33 (54.1%)	21 (70.0%)	55 (74.3%)
Lymph nodes	92 (35.7%)	36 (38.7%)	26 (42.6%)	14 (46.7%)	16 (21.6%)
Lung	78 (30.2%)	25 (26.9%)	15 (24.6%)	15 (50.0%)	23 (31.1%)
Liver	66 (25.6%)	17 (18.3%)	20 (32.8%)	12 (40.0%)	17 (23.0%)
Breast	40 (15.5%)	18 (19.4%)	13 (21.3%)	7 (23.3%)	2 (2.7%)
Pleura	15 (5.8%)	6 (6.5%)	2 (3.3%)	2 (6.7%)	5 (6.8%)
Brain	12 (4.7%)	4 (4.3%)	1 (1.6%)	2 (6.7%)	5 (6.8%)
Chest wall	4 (1.6%)	1 (1.1%)	1 (1.6%)	0	2 (2.7%)
Other	33 (12.8%)	13 (14.0%)	12 (19.7%)	3 (10.0%)	5 (6.8%)
Missing/NA	9 (3.5%)	5 (5.4%)	2 (3.3%)	0	2 (2.7%)
Visceral (*n* [%])^b^	136 (52.7%)	45 (48.4%)	30 (49.2%)	22 (73.3%)	39 (52.7%)
Nonvisceral (*n* [%])	223 (86.4%)	80 (86.0%)	51 (83.6%)	26 (86.7%)	66 (89.2%)
Bone only (*n* [%])	107 (41.5%)	34 (36.6%)	17 (27.9%)	11 (36.7%)	45 (60.8%)
Prior endocrine treatment (*n*)	165	34	49	23	59
Endocrine sensitivity at initiation of palbociclib (*n* [%])^§^					
Sensitive	46 (17.8%)	28 (30.1%)	11 (18.0%)	6 (20.0%)	1 (1.4%)
Primary resistant	16 (6.2%)	1 (1.1%)	14 (23.0%)	0	1 (1.4%)
Secondary resistant	10 (3.9%)	2 (2.2%)	5 (8.2%)	3 (10.0%)	0
Unknown	51 (19.8%)	25 (26.9%)	10 (16.4%)	9 (30.0%)	7 (9.5%)
Missing/NA	135 (52.3%)	37 (39.8%)	21 (34.4%)	12 (40.0%)	65 (87.8%)
Initiation time of palbociclib treatment (relative to the first BC diagnosis) (years)					
Mean (SD)	4.7 (4.65)	2.5 (3.72)	4.5 (3.69)	6.6 (5.19)	6.9 (4.98)
Median	3.4	1.3	3.4	4.6	5
IQR	(1.3, 6.4)	(0.6, 2.5)	(1.6, 6.4)	(2.6, 9.3)	(3.7, 8.7)
Min, Max	0.1, 23.6	0.1, 22.2	0.1, 15.6	1.5, 19.2	0.4, 23.6
Missing/NA	51	23	6	2	20
Duration of follow‐up (months)					
Mean (SD)	19.2 (11.28)	20.4 (11.76)	16.8 (11.64)	19.2 (10.68)	19.2 (10.68)
Median	16.8	20.4	12.0	16.8	19.2
IQR	(9.6, 26.4)	(9.6, 27.6)	(7.2, 25.2)	(9.6, 25.2)	(10.8, 26.4)
Min, Max	0, 60	0, 60	0, 48	0, 48	0, 48
Missing/NA	1	0	0	0	0

*Note:* Percentages are based on the number of eligible patients in each column; percentages for early BC and ABC are based on the number of eligible patients in each group, respectively. Median, Min, and Max are not applicable if *n* < 2.

Abbreviations: 1L, first‐line; 2L, second‐line; 3L+, third‐line and beyond; ABC, advanced breast cancer; BC, breast cancer; BMI, body mass index; EBC, early breast cancer; ECOG, Eastern Cooperative Oncology Group; ET, endocrine therapy; IQR, interquartile range; Max, maximum; Min, minimum; n, number of patients; NA, not available; SD, standard deviation.

^§^Endocrine sensitive: Patients who had never received ET for EBC, those who relapsed ≥ 12 months after completion of adjuvant ET, or those diagnosed with de novo stage IV BC. Primary endocrine‐resistant disease: Relapse while on the first 2 years of adjuvant ET, or disease progression within the first 6 months of 1L ET for advanced BC while on ET. Secondary (acquired) resistance: Relapse while on adjuvant ET but after the first 2 years, or relapse within 12 months of completing adjuvant ET, or disease progression ≥ 6 months after initiating ET for advanced BC while on ET.

^a^Multiresponse.

^b^Nonvisceral metastases were defined as lesions in bone, soft tissue, lymph nodes, chest wall, and/or skin. Visceral metastases were defined as any other lesion location.

### 3.3. Treatment Patterns

Regardless of the treatment regimen, the majority of patients (96.5%, *n* = 249) followed a schedule consisting of 3 weeks on therapy followed by 1 week off. The recommended 125‐mg dose was prescribed as the initial dose to 88.4% (*n* = 228) of the patients. Twenty‐nine patients initiated treatment at lower doses (100 mg, 10.5%, *n* = 27; and 75 mg, 0.8%, *n* = 2; dose unknown for one patient).

A total of 91 patients (35.3%) experienced cycle interruptions, with laboratory abnormalities being the main cause in 65.9% (*n* = 60) of the patients. Most patients (*n* = 166, 64.3%) did not undergo any modifications to their dosing or treatment schedule. Sixty‐two patients (24.0%) experienced dose modifications, 9 patients (3.5%) experienced schedule changes outside the indicated 3 weeks on/1 week off, and 7 patients (2.7%) had both dose and schedule changes.

The most common reason for the dose and/or schedule modifications was to manage toxicity (*n* = 53; 68.0%). Cycle delays were reported in 83 patients (32.2%), and laboratory abnormalities were the reason for delays in three‐quarters of the cases. Information regarding palbociclib treatment management is presented in Table 2.

**Table 2:** Descriptive summary of palbociclib treatment—overall and by line of treatment.

Variable	Line of treatment				
	Overall	1L	2L	3L+	Missing
Receiving palbociclib (% of total cohort)	258 (100%)	93 (36.0%)	61 (23.6%)	30 (11.6%)	74 (28.7%)
Treatment schedule “3 weeks on, 1 week off” (*n* [%])					
Yes	249 (96.5%)	90 (96.8%)	61 (100.0%)	29 (96.7%)	68 (91.9%)
No	3 (1.2%)	1 (1.1%)	0	1 (3.3%)	1 (1.4%)
Missing	6 (23.3%)	2 (2.2%)	0	0	5 (6.8%)
Starting dose (*n* [%])					
125 mg	228 (88.4%)	76 (81.7%)	58 (95.1%)	27 (90.0%)	67 (89.2%)
100 mg	27 (10.5%)	16 (17.2%)	3 (4.9%)	3 (10.0%)	5 (6.8%)
75 mg	2 (0.8%)	0	0	0	2 (2.7%)
Missing	1 (0.4%)	1 (1.4%)	0	0	0
Reasons starting dose < 125 mg (*n* [%])					
*n* pts with reduced starting dose	29	16	3	3	7
To avoid toxicity	17 (58.6%)	10 (62.5%)	3 (100%)	1 (33.3%)	3 (42.9%)
Age	1 (3.4%)	1 (6.3%)	0	0	0
Disease stage	1 (3.4%)	0	0	1 (33.3%)	0
Other	1 (3.4%)	1 (6.3%)	0	0	0
Missing	9 (31.0%)	4 (25.0%)	0	1 (33.3%)	4 (57.1%)
Accompanying endocrine treatment (*n* [%])					
Yes	256 (99.2%)	92 (98.9%)	60 (98.4%)	30 (100.0%)	74 (100.0%)
No	1 (0.4%)	0	1 (1.6%)	0	0
Missing	1 (0.4%)	1 (1.1%)	0	0	0
Type of endocrine treatment (*n* [%])^a^					
Letrozole	131 (50.8%)	68 (73.1%)	29 (47.5%)	6 (20.0%)	28 (37.8%)
Fulvestrant	120 (46.5%)	23 (24.7%)	33 (54.1%)	24 (80.0%)	40 (54.1%)
Exemestane	10 (3.9%)	3 (3.2%)	1 (1.6%)	2 (6.7%)	4 (5.4%)
Tamoxifen	2 (0.8%)	0 (0.0%)	0 (0.0%)	0 (0.0%)	2 (2.7%)
Anastrozole	1 (0.4%)	0 (0.0%)	1 (1.6%)	0 (0.0%)	0 (0.0%)
Cycle interruption (*n* [%])					
No	150 (58.1%)	61 (65.6%)	44 (72.1%)	18 (60.0%)	27 (36.5%)
Yes	91 (35.3%)	26 (27.5%)	15 (24.6%)	8 (26.7%)	42 (66.6%)
Missing	17 (6.6%)	6 (6.5%)	2 (3.3%)	4 (13.3%)	5 (6.8%)
Most frequent reasons for cycle interruption (*n* [%])^b$^					
*n* pts cycle with cycle interruption	91	26	15	8	42
Lab abnormalities	60 (65.9%)	14 (53.8%)	8 (53.3%)	3 (37.5%)	35 (83.3%)
Other	29 (31.9%)	13 (50.0%)	7 (46.7%)	2 (25.0%)	7 (16.7%)
Missing	0	0	0	0	0
Modification in dose and/or schedule (*n* [%])					
No modification	166 (64.3%)	66 (71.0%)	43 (70.5%)	18 (60.0%)	39 (52.7%)
Dose	62 (24.0%)	16 (17.2%)	14 (23.0%)	7 (23.3%)	25 (33.8%)
Schedule	9 (3.5%)	4 (4.3%)	2 (3.3%)	0 (0.0%)	3 (4.1%)
Dose and schedule	7 (2.7%)	1 (1.1%)	1 (1.6%)	0 (0.0%)	5 (6.8%)
Missing	14 (5.4%)	6 (6.5%)	1 (1.6%)	5 (16.7%)	2 (2.7%)
Most frequent reasons for dose and/or schedule modification (*n* [%])^b#^					
*n* pts with modification	78	21	17	7	33
To manage toxicity	53 (68.0%)	12 (57.1%)	12 (70.6%)	2 (28.6%)	27 (81.8%)
Other	18 (23.1%)	8 (38.1%)	4 (23.5%)	1 (14.3%)	5 (15.2%)
Missing	3	1	0	2	0
Cycle delays (*n* [%])					
No	162 (62.8%)	64 (68.8%)	46 (75.4%)	19 (63.3%)	33 (44.6%)
Yes	83 (32.2%)	26 (28.0%)	15 (24.6%)	8 (26.7%)	34 (45.9%)
Missing	13 (5.0%)	3 (3.2%)	0 (0.0%)	3 (10.0%)	7 (9.5%)
Most frequent reasons for cycle delays (*n* [%])^b&^					
*n* pts with cycle delays	83	26	15	8	34
Lab abnormalities	62 (74.7%)	20 (76.9%)	13 (86.7%)	3 (37.5%)	26 (76.5%)
Adverse events	7 (8.4%)	0	1 (6.7%)	1 (12.5%)	5 (14.7%)
Other	14 (16.9%)	4 (15.4%)	4 (26.7%)	3 (37.5%)	3 (8.8%)
Missing	0	2	0	0	0

Abbreviations: 1L, first‐line; 2L, second‐line; 3L+, third‐line and beyond; ABC, advanced breast cancer; BC, breast cancer; EBC, early breast cancer; ECOG PS, Eastern Cooperative Oncology Group Performance Status; IQR, interquartile range; *n*, number of patients; NA, not available; SD, standard deviation.

^$^Additional reasons for cycle interruptions included patient request (*n* = 2), worsening of ECOG PS (*n* = 1), comorbidities (*n* = 1), and concomitant medications (*n* = 1).

^#^Additional reasons for dose and/schedule modification included line of treatment (*n* = 1), ECOG PS (*n* = 1), patient request (*n* = 1), and metastases status (*n* = 1).

^&^Additional reasons for cycle delays included comorbidities (*n* = 1), patient request (*n* = 1), and concomitant medications (*n* = 1).

^a^Patients may have received more than one medication.

^b^Multiple reasons per patient.

In the 1L setting, 73.1% (*n* = 68/93) of patients received palbociclib + an AI, whereas 24.7% (*n* = 23/93) received palbociclib + fulvestrant. In the 2L setting, 47.5% (*n* = 29/61) of patients received palbociclib + AI, whereas 54.1% (*n* = 33/61) received palbociclib + fulvestrant. Most 3L patients (80.0%, *n* = 24/30) received palbociclib + fulvestrant, whereas 20.0% (*n* = 6/30) received palbociclib + AI (Table 2).

### 3.4. Clinical Outcomes

**Figure 2:**
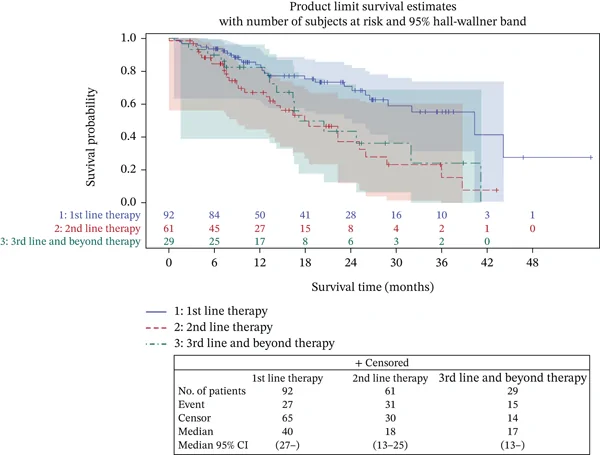
rwPFS by line of treatment following palbociclib treatment initiation. Abbreviation: CI, confidence interval.

Outcome data were available for 182 patients (Figure 1). Of these, 92 patients (50.5%) were in 1L, 61 (33.5%) in 2L, and 29 (15.9%) in 3L+. Median overall rwPFS was 26.5 months (1L: 40.3 months; 2L: 18.5 months; 3L: 17.2 months). The 12‐, 24‐, and 36‐month rwPFS rates by line of treatment were 84.5%, 72.8%, and 58.4% for 1L; 69.8%, 38.8%, and 16.2% for 2L; 80.0%, 42.3%, and 23.5% for 3L+ (Table 3 and Figure 2).

**Table 3:** Clinical outcomes of palbociclib treatment—overall and by line of treatment.

		Line of treatment		
Variables	Overall	1L	2L	3L+
**Receiving palbociclib (% of patients with available outcome data)**	182 (100%)	92 (50.5%)	61 (33.5%)	29 (15.9%)
**rwPFS rates (Kaplan–Meier analysis) (% [95% CI])**				
At 12 months	79.2% (73.1, 85.3)	84.5% (76.9, 92.1)	69.8% (57.6, 82.1)	80.0% (65.6, 94.4)
At 24 months	58.0% (49.3, 66.7)	72.8% (62.3, 83.3)	38.8% (21.7, 55.8)	42.3% (20.2, 64.3)
At 36 months	40.1% (29.1, 51.1)	58.4% (44.1, 72.7)	16.2% (1.0, 33.3)	23.5% (0.5, 47.5)
**Median rwPFS (Kaplan–Meier analysis) (month)**	26.5	40.3	18.5	17.2
**Overall tumor response** ^**a**^				
*n*	182	92	61	29
Complete response	18 (9.9%)	14 (15.2%)	4 (6.6%)	0
Partial response	48 (26.4%)	29 (31.5%)	12 (19.7%)	7 (24.1%)
Progressive disease	14 (7.7%)	3 (3.3%)	7 (11.5%)	4 (13.8%)
Stable disease	68 (37.4%)	28 (30.4%)	30 (49.2%)	10 (34.5%)
Unknown	4 (2.2%)	2 (2.2%)	1 (1.6%)	1 (3.4%)
Missing	30 (16.5%)	16 (17.4%)	7 (11.5%)	7 (24.1%)

*Note:* Percentages are based on nonmissing outcome observations. Median duration of follow‐up was 16.8 months overall and 20.4 months in 1L, 12.0 months in 2L, and 16.8 months in 3L+, respectively (follow‐up duration by LOT is presented in Table 1).

Abbreviations: 1L, first‐line; 2L, second‐Line; 3L+, third‐line and beyond; ABC, advanced breast cancer; BC, breast cancer; BMI, body mass index; EBC, early breast cancer; ECOG, Eastern Cooperative Oncology Group; IQR, interquartile range; LOT, line of treatment; *n*, number of patients; NA, not available; rwPFS, real‐world progression‐free survival; SD, standard deviation.

^a^Based on unconfirmed best overall response.

OS data were immature at the time of analysis given the median follow‐up of 16.8 months and are presented in Table S1.

Among the patients with available clinical outcome data, complete response (CR) was reported in 18 patients (9.9%), partial response (PR) was achieved by 48 patients (26.4%), and 14 patients (7.7%) had progressive disease. Palbociclib showed higher response rates in first‐line treatment, with CR at 15.2% and PR at 31.5%, compared with lower rates in later lines (CR: 6.6%, 0%; PR: 19.7%, 24.1%).

## 4. Discussion

The TREASURE study investigated palbociclib treatment patterns and outcomes among 258 patients with HR+/HER2− ABC across 7 Gulf region sites, offering valuable regional insights. Our data demonstrated that the use of palbociclib in ABC was associated with favorable outcomes and generally well tolerated, with 88.4% of patients starting at 125 mg and only 24.0% requiring dose modifications—lower than the 34%–36% reported in PALOMA‐2 and PALOMA‐3, suggesting potential regional differences in prescribing behaviors [19, 21]. In line with other RWE studies of palbociclib, bone was the most frequent metastatic site (41.5%), mirroring the IRIS real‐world study (43.5%) but higher than in the PALOMA‐2 clinical trial (23%) [26, 27, 30, 37, 41, 42].

Nonetheless, we identified notable demographic differences: Our cohort′s median age (53.7 years) was younger than the ~58–64 years often noted in other RWE studies [37, 43–46], and the proportion of premenopausal patients (39.2%) exceeded the 12.2% observed in the IRIS real‐world study (palbociclib real‐world study involving 12 countries across North America, South America, and Europe) [38, 47, 48, 39]. Such differences likely reflect local epidemiologic patterns in the Gulf region, where BC often manifests earlier. These findings confirm the broader applicability of palbociclib in HR+/HER2− ABC while underscoring the distinctive demographic profile of younger premenopausal patients in this setting.

Median rwPFS in the present cohort was 26.5 months overall, 40.3 months in 1L, 18.5 months in 2L, and 17.2 months in 3L+. The 1L and 2L values are higher than the median PFS reported in PALOMA‐2 (24.8 months for palbociclib plus letrozole) and PALOMA‐3 (9.5 months for palbociclib plus fulvestrant), respectively. Real‐world studies of palbociclib have reported PFS estimates that span a wide range, in part because real‐world cohorts include patients who would not have met the eligibility criteria for the pivotal trials [38]. In the systematic scoping review by Rauthan et al. [38], median PFS for 1L palbociclib plus ET in countries underrepresented in clinical trials ranged from 20.2 to 36.7 months, and from 7.0 to 24.2 months in 2L or later settings. In the systematic review and meta‐analysis by Schettini et al. [47] of 12 real‐world palbociclib studies, the pooled 1L median rwPFS was 22.5 months for palbociclib plus AI, in line with the PALOMA‐1/2 pooled estimate, and 13.5 months for palbociclib plus fulvestrant, above the PALOMA‐3 estimate. In the multinational European study by Oikonomidou et al. [48] of 856 patients receiving 1L palbociclib plus AI, median rwPFS was 28.1 months in Spain, 33.9 months in the United Kingdom, and 48.1 months in Germany. The 1L value in the present cohort lies at the upper end of these published ranges, slightly above the Rauthan upper bound and within the Oikonomidou range. The methodological factors discussed in the Limitations section, including the absence of standardized response criteria such as RECIST, the substantial proportion of missing data on key baseline variables, and the retrospective design, may have influenced the rwPFS estimates and contributed to longer values relative to the pivotal trials. The similar median rwPFS in 2L (18.5 months) and 3L+ (17.2 months) likely reflects the small subgroup sizes for outcome analysis (2L *n* = 61, 3L + *n* = 29).

In our study, 12‐, 18‐, and 24‐month rwPFS rates for the whole cohort were 79.2%, 58.0%, and 40.1%, respectively. These results are consistent with findings reported in other RWE studies [37, 43–46]. Notably, our study reported higher rwPFS at 12 months compared with those reported by Al‐Foheidi et al. [39], which were derived from data collected at 2 cancer centers in Saudi Arabia, where rwPFS was 58%. The variation between our study and the study by Al‐Foheidi et al. may be explained by the larger percentage of patients in their cohort who had extensive prior treatment, with 55% receiving previous systemic chemotherapy and 49% undergoing 2 or more lines of ET before initiating palbociclib. In contrast, our study included a larger proportion of 1L patients (36.0%), who typically exhibit longer rwPFS and OS compared with later line patients. Additionally, differences in treatment management, disease burden, and real‐world progression assessment may have contributed to the observed variation in outcomes.

In a more recent retrospective Egyptian cohort, Ebid et al. [40] studied 140 patients with endocrine‐resistant HR+/HER2− metastatic breast cancer treated with palbociclib plus ET. OS was longer in patients with secondary than with primary endocrine resistance and in patients who received palbociclib for at least 12 months. Taken together, the present findings and the data from Al‐Foheidi et al. and Ebid et al. [40] indicate that real‐world outcomes of palbociclib in the MENA region are influenced by line of therapy, endocrine sensitivity, and prior treatment burden. This study outlines the treatment patterns and outcomes of palbociclib in the region; however, it also identifies certain limitations that could have affected the results and may impact their generalizability. Firstly, our retrospective, descriptive design and relatively small sample pooled from three countries likely do not capture the full heterogeneity of Gulf populations. In addition, this study assessed palbociclib across multiple lines of treatment, although the patient numbers in each cohort were limited. Secondly, missing data was substantial for several variables, particularly for ECOG performance status, endocrine sensitivity, and comorbidities. These gaps reduced the robustness of subgroup analyses and contributed to variability in some baseline characteristics, and they should be taken into account when interpreting the present findings. Comparable levels of incompleteness on key baseline variables have been reported in retrospective real‐world studies based on electronic health record data, including in established healthcare systems [49]. Although this does not mitigate the impact on our results, it indicates that data incompleteness is a recurring feature of real‐world chart‐review studies and one that is often not quantified in published reports.

Tumor response and disease progression were assessed by treating physicians in routine clinical practice rather than according to standardized criteria such as RECIST or at prespecified time points, and measurable disease was not formally assessed; these methodological features, inherent to noninterventional studies, may have affected the interpretation of the rwPFS estimates. The analysis dataset did not include the specific agents of prior ET, the setting and regimens of prior chemotherapy, or the number of metastatic sites per patient, and these data are not reported. Specific adverse events, laboratory abnormalities, treatment discontinuations attributed to specific adverse events, and anticancer therapies received after palbociclib discontinuation or switching were not collected and are not reported.

Finally, variations in health care systems, clinical practices, and genetic backgrounds across the region may have also influenced outcomes. Nevertheless, these results provide relevant real‐world data on palbociclib treatment, addressing a gap in HR+/HER2− ABC management evidence from the region.

## 5. Conclusion

The TREASURE study conducted in the Arabian Gulf region offers valuable insights into patient characteristics, treatment patterns, and clinical outcomes of patients with HR+/HER2− ABC receiving palbociclib. These findings are relevant for understanding the management of patients treated with palbociclib in this part of the world. Further research involving larger cohorts and extended follow‐up durations is warranted to more accurately assess long‐term clinical outcomes in this patient population.

## Author Contributions

A.A. and A.H.: conceptualization and methodology; E.D., A.A.F., N.A., S.K., M.F.L., M.O., S.T., S.D., and M.A‐F.: data collection and validation; all authors: writing—original draft—review and editing.

## Funding

This study was supported by Pfizer (10.13039/100004319).

## Disclosure

The authors attest that Pfizer has had no influence on design of this study or its outcomes.

## Ethics Statement

This study was approved by the following institutional review boards/ethics committees (Ethics Approval Code): Dubai Hospital DHA (DSREC‐09/2020_12), Ministry of National Guard Health Affairs‐Jeddah (IRBC/1376/20), Ministry of National Guard Health Affairs‐Riyadh (IRBC/1130/20), Hamad Medical Corporation (MRC‐02‐20‐261), King Saud University (20/0699/IRB), Mediclinic (MCME.CR.100.MCIT.2020), and Tawam Hospital (DOH/CVDC/2021/1511). The study was conducted in accordance with legal and regulatory requirements, as well as with scientific purpose, value, and rigor, and followed generally accepted research practices described in Good Pharmacoepidemiological Practice, the Declaration of Helsinki, and applicable national guidelines and Good Pharmacovigilance Practices. As this study did not involve data subject to privacy laws according to applicable legal requirements, obtaining informed consent from patients was waived.

## Conflicts of Interest

Authors A.A. and A.H. are affiliated with and funded by Pfizer (employment). E.D. is affiliated with Tawam Hospital. F.C. and A.A.F. are affiliated with Hamad Medical Corporation. N.A. is affiliated with National Guard Hospital (Riyadh). S.K. is affiliated with King Saud University. M.F.L., M.O., and S.T. are affiliated with Dubai Hospital. S.D. is affiliated with Mediclinic Hospital. M.A‐F. is affiliated with National Guard Hospital (Jeddah).

## Supporting information


**Supporting Information** Additional supporting information can be found online in the Supporting Information section. Table S1: The overall survival rate at 12 and 24 months in all patients and by line of treatment.

## Data Availability

The data that support the findings of this study are not publicly available due to privacy or ethical restrictions.
