# Supplementary material for: Treatment Patterns and Clinical Outcomes Among Patients Receiving Palbociclib Combinations for Hormone Receptor–Positive (HR+)/Human Epidermal Growth Factor Receptor 2–Negative (HER2−) Advanced/Metastatic Breast Cancer in the Arabian Gulf Region: A Retrospective Study (TREASURE Study)
Source: Int J Breast Cancer. 2026 Sep 22;2026:3477817. doi: 10.1155/ijbc/3477817 (PMC13598375; doi:10.1155/ijbc/3477817)
Supplement: Supplementary file 1 [file IJBC-2026-3477817-s001.docx]

**Supplementary Tables**

**Supplementary Table S1: Overall survival at 12 and 24 months — All patients and by line of treatment**

| **Patients alive (Kaplan–Meier analysis) (% [95% CI])**** | | | | |
| --- | --- | --- | --- | --- |
|  |  | **Line of treatment** | | |
|  | **All patients** | **1L** | **2L** | **3L+** |
| At 12 months | 94.0%  (90.2, 97.9) | 96.3%  (92.1, 100.5) | 87.4%  (77.7, 97.0) | 100.0%  (100.0, 100.0) |
| At 24 months | 83.7%  (76.7, 90.8) | 86.0%  (76.7, 95.4) | 76.6%  (63.5, 89.7) | 91.7%  (76.0, 100) |

**Included all the enrolled patients, except one patient with date of palbociclib treatment missing.
